# Sensitivity and specificity analysis of 2D small field measurement array: Patient‐specific quality assurance of small target treatments and spatially fractionated radiotherapy

**DOI:** 10.1002/acm2.13402

**Published:** 2021-08-27

**Authors:** Maria Carmen Banos‐Capilla, Jose Domingo Lago‐Martin, Patricia Gil, Luis Maria Larrea

**Affiliations:** ^1^ Radiation Oncology Department Hospital Vithas Consuelo Valencia Spain; ^2^ Mathematical and Fluid Physics Department Faculty of Sciences National University of Distance Education (UNED) Madrid Spain; ^3^ Radiation Oncology Department Fundación instituto Valenciano de Oncología Valencia Spain

**Keywords:** diode arrays, dosimetry, IMRT QA, small‐field dosimetry, SRS‐resolution arrays

## Abstract

**Purpose:**

The aim of this paper is to describe the tests carried out on a SRSMapCheck array, to verify its reliability and sensitivity for quality assurance (QA) of high gradient treatments as an alternative system to the use of high spatial resolution detectors, such as gafchromic film, whose processing requires meticulous and time‐consuming procedures.

**Methods:**

In an initial step, general functionality tests were carried out to verify that the equipment meets the manufacturer's specifications. A study of the accuracy of the application of correction factors to compensate for variation in detector response due to dose rate, field size and beam angle incidence has been included. Besides, to assess the ability of the array to detect inaccurately delivered treatments, systematic errors corresponding to the deviation in the position of the leaves and the accuracy of the gantry position, have been introduced. Based on these results, an estimate of sensitivity and specificity values of the device has been completed. The final step included a study applied to high gradient treatment for real cases of spatially fractionated radiotherapy, where the results of SRSMapCheck measurements have been compared with gafchromic films.

**Results:**

General commissioning tests meet the manufacturer's specifications. dose rate (DR) response variation is better than 1.5% and for DR above 50 MU/min better than 1%. The results for beam incidences are better than 1% for all gantry angles, including beam incidences parallel to the array. Field size response differences are within the range of ±1% for sizes up to 2 × 2 cm^2^, with a maximum value obtained of 3.5%, for 1 × 1 cm^2^. From the systematic error study, using a Gamma function Γ (2%, 2 mm), the detector presents a high specificity with a value greater than 90% at its lower limit, while its sensitivity has a moderate mean value of 81%. Sensitivity values increase above 86% when we apply a Gamma function Γ (2%, 1 mm) is applied. Finally, the study of actual cases comprises 17 patients, distributed into 11 lung tumors, 3 gynecological and 3 soft tissue tumors. The gafchromic film showed a lower passing rate with an average value of Γ (2%, 2 mm) = 94.1% compared to Γ (2%, 2 mm) = 98.6% reached by the measurements with the array.

**Conclusions:**

Gamma function obtained with the SRSMapCheck array always presented a higher value than gafchromic film measurements, resulting in a greater number of plans considered correct. This fact, together with the sensitivity and specificity study carried out, allows us to conclude the recommendation that a restrictive metric must be established, in this way we will improve sensitivity, and therefore we will reduce the rate of incorrect plans qualified as correct. The characteristics of the equipment together with the correction factors applied, led to reliably performing acquisitions for complex treatments with multiple small targets in oblique rotational incidences. The spatial resolution of detectors allows the verification of high gradient dose plans such as those achieved in spatially fractionated radiotherapy (SFRT).

## 1 INTRODUCTION

The incorporation of the intensity modulated radiotherapy (IMRT) has allowed the development of high level complex new treatment techniques. Among these challenging treatments spatially fractionated radiotherapy (SFRT) is a technique that applies a dose boost through a series of multiple targets distributed within the gross tumor volume (GTV), while concomitantly, the dose to the planning target volume (PTV) is delivered in a conventional fractionation.^1^, ^2^ High dose levels inside bulky tumors allow achieving a fast volume reduction thanks to the bystander and abscopal radiobiological effects.^1^, ^3^ The degree of modulation necessary to reach the high dose gradients applied to the multiple targets is generated by several small beamlets whose configuration is obtained through inverse calculation algorithms. This highly modulated dosimetry requires careful verification of deliverability before delivering patient treatment.^4^, ^5^

Given treatment complexity, the verification of beam control parameters included in the quality assurance (QA) protocols does not ensure by itself the deliverability of planned treatment.^4^, ^5^, ^6^ A specific analysis of dose accuracy prior to treatment application is necessary to ensure the final quality of the treatments. These tests are usually performed using high spatial resolution detectors such as gafchromic film or polymer gels, whose processing requires meticulous and time‐consuming practices. The 2D‐Arrays are a good alternative to traditional verification methods, as they make verification more efficient while decreasing processing time. Several manufacturers’ firms have presented high‐resolution 2D‐array detectors specifically designed for the verification of treatment with a high‐level of complexity. Among these 2D‐array solutions, the one we have chosen for our study corresponds to the SRSMapCheck model from Sun Nuclear Corporation, Melbourne, FL, USA.

If we focus on equipment characteristics, high modulation IMRT techniques involve situations that push array detectors to the limit of their functionality. The response stability of the detectors to the variation of different dosimetric parameters must be thoroughly studied before the results of the verification of patient treatments can be accepted. Lack of lateral electronic equilibrium in small radiation fields, dose‐per‐pulse response or beam incidence are some of the factors to be studied and whose results should be contrasted with those obtained by other published authors. It is also necessary to establish the reliability limits of the equipment, both related to the array resolution and to the variation in the response of each individual diode to high and steep gradient dose fields.^7^

The aim of this paper is to describe the tests carried out on a high spatial resolution 2D array, model SRSMapCheck (Sun Nuclear Corp., Melbourne, FL), to verify its reliability, specificity and sensitivity to detect misalignments and dosimetric errors. Moreover, a comparative study with a higher resolution detection system will be carried out to verify its reliability to be used for patient specific QA, for high gradient treatments such as SFRT.

## 2 MATERIAL

The QA patient specific tests comprise measurement of absolute dose at discrete points with an ionization chamber (IC) and a comparative study of fluence maps. For high accuracy point measurements, the use of small‐volume IC is recommended. To obtain absolute dose distribution differences we have compared the fluence maps obtained from the array of our study to those calculated by the radiotherapy planning system (RTPS) and we have also compared the results obtained, following the same methods but employing an alternative high‐resolution method whose results are highly contrasted, the gafchromic film.

### 2.1 Spatially fractionated radiotherapy

SFRT treatments are high‐complexity treatments performed with a VMAT/IMRT technique, which conforms the dose to a high‐volume PTV, while modulating the dose in small segments, a high‐level dose is required to generate multiple small diameter targets spread within the PTV. These treatments are of a complexity equivalent to multiple target SRS treatments of small size metastases treated with one isocenter. So, SFRT treatments combine two treatment types: on the one side, the dose should cover and conform to an extensive PTV, for which both the sizes and degree of modulation do not require a special or different detector than commonly used in IMRT treatments. And on the other side, these same VMAT/IMRT beams that conform to the PTV, modulate by means of small segments, the high dose levels within the multiple 1 cm diameter targets within the PTV.

**FIGURE 1:**
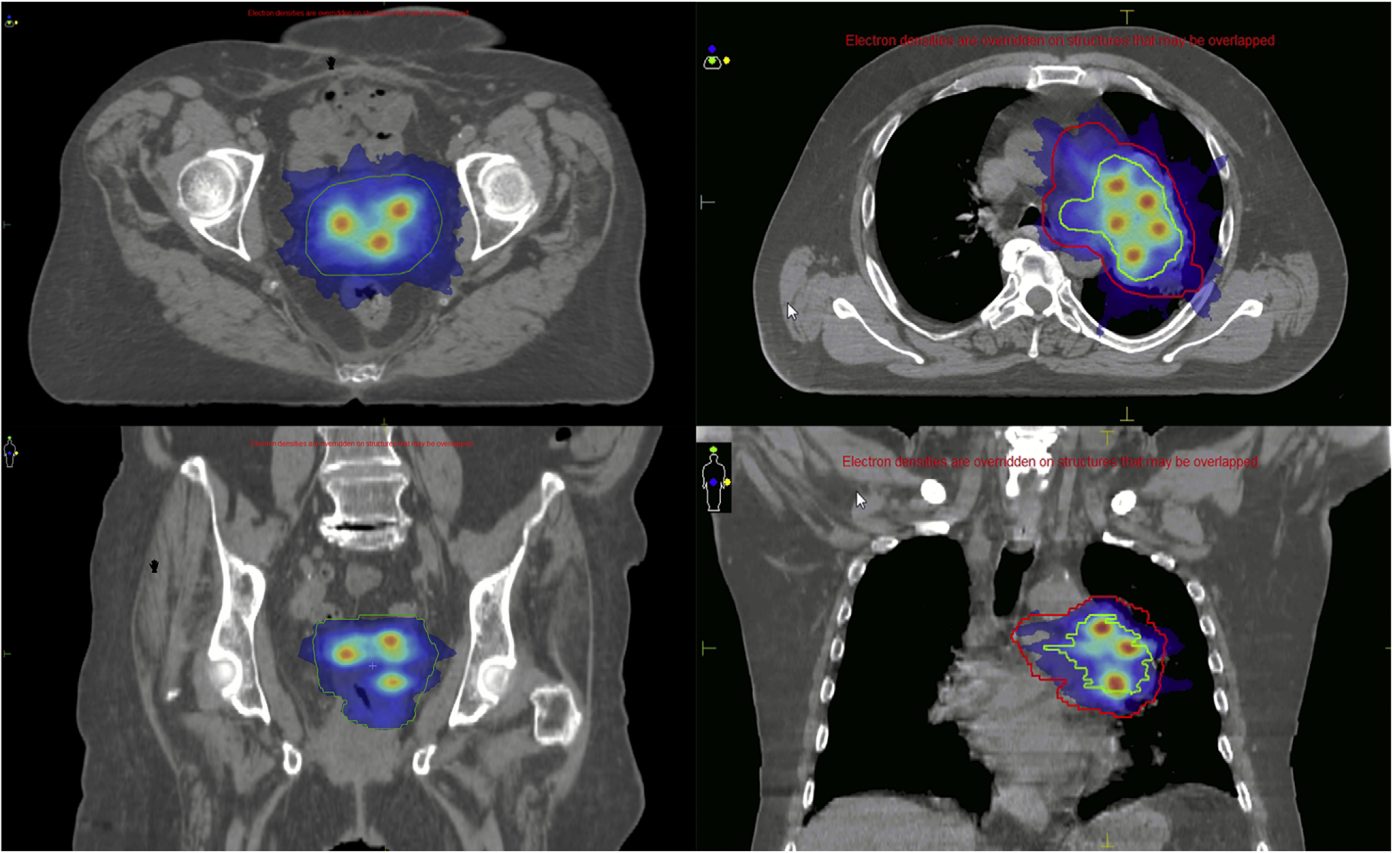
One isocenter but multiple targets prescribed inside the GTV delivered at the same time as the conventional fraction treatment of the PTV. Example of two SFRT dose distributions: Axial and coronal planes of gynecological (left) and lung tumor (right) treatments

Some examples of dose distribution are shown in Figure 1, where it is observed how the boost dose is administered over at least 5 targets in the shape of small spheres distributed within the GTV as the dose conforms to surround the PTV and to preserve the surrounding healthy tissues.

### 2.2 Treatment unit and planning system

Treatment planning calculations have been made with Elekta's MONACO v5.1.11 RTPS using a Monte Carlo XVMC algorithm, with a 1 mm calculation grid and 0.5% of statistical uncertainty. The SFRT treatments have been calculated using volumetric IMRT techniques (VMAT). Two non‐coplanar double arcs with a single isocenter have been used to generate plans.

The treatment unit employed was a 6 MV Infinity Linac from Elekta. This linear accelerator has a multileaf collimator model Agility whose leaf size is 5 mm at the isocenter and the maximum travel speed reaches 65 mm/s. Our linac has no flattening filter free (FFF) function available, so the maximum dose rate achieved is about 600 MU/min.

### 2.3 Ionization chamber

The specific patient verification protocol begins with point absolute dose measurements performed with a small‐volume ionization chamber, a PTW model 31006 pin‐point type thimble ionization chamber was used; this IC has a sensitive volume of 0.015 cm^3^, a diameter of 2.0 mm and a 4.0 mm length.^8^ The dose calculated at a point of measurement, located in a low‐gradient area, has been obtained by means of an average dose value over a volume equivalent to the sensitive volume of the chamber.^8^ Following the recommendations from the bibliography,^4^ a cross‐measurement was carried out for a reference field of 5 × 5 cm^2^, with a 0.6 cm^3^ PTW Farmer‐type ionization chamber calibrated at an accredited dosimetry calibration laboratory (ADCL). The ionization measurements have been completed within a spherical polymethyl methacrylate (PMMA) phantom designed for end‐to‐end tests, Model Lucy 3D QA by standard imaging.

### 2.4 Gafchromic film

Gafchromic EBT3 (Ashland, USA)^9^ Film was used to contrast results in isodose distributions. The dose dynamic range of the film is 0.1  to 20 Gy but following the manufacturer's specifications for the gafchromic EBT3, optimum results are obtained when the maximum dose applied is less than 10 Gy. We have performed the calibration of the film over its dynamic dose range (0.1  to 20 Gy) following the usual calibration procedure described in the published protocols, TG55‐TG235 AAPM.^10^, ^11^ It has been scanned to 48bit with a 72dpi resolution, always regarding the same direction with respect to the longitudinal axis of the scanner (Epson Expression 12000XL). A fiducial set of points establishes the treatment isocenter position to aid the registration process and maintain the orientation relative to the scanner's long axis. Scanned images have been processed with the software OmniPro‐ImRT v1.7.0021 (IBA Dosimetry, Germany), where the dosimetric information corresponding to the red channel is extracted and the calibration curve is applied. In this application, film images have been qualitatively contrasted with the RTPS exported fluence maps by overlapping both the isodose planes and the profiles along the main axis. The quantitative analysis has been performed based on the Gamma function^4^, ^12^ calculated with different metrics.

### 2.5 2D array

**FIGURE 2:**
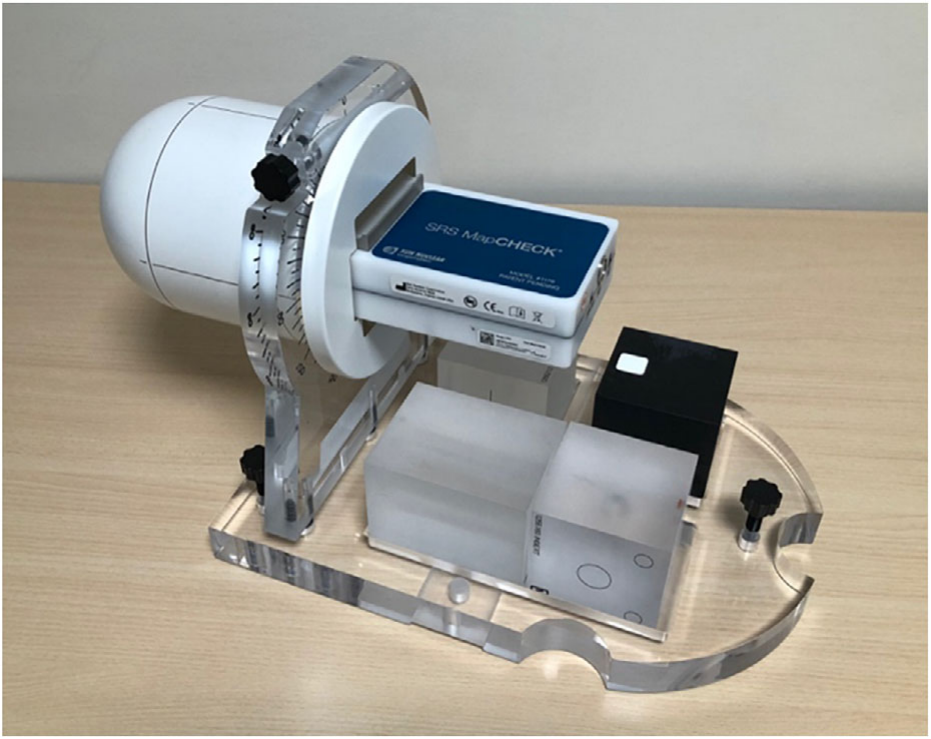
Picture of the SRSMapCheck array placed inside the StereoPHAN. The phantom allows the array to turn around the longitudinal axis

The equipment selected for the test was an SRS 2D‐array, model SRSMapCheck whose acquisition and processing measurement software was SNC (Sun Nuclear Corp., Melbourne, FL).^13^ The array consists of a distribution of 1013 diodes of 0.48 mm × 0.48 mm cross‐section, with their centers spaced 2.47 mm apart. The active area covered by the diodes is 77 × 77 mm^2^. The diodes are distributed on two overlapped printed circuit boards (PCBs) and aligned so that the active pn‐junctions lay on the same plane. Around these PCBs, a 2.25 cm PMMA layer acts as a buildup and backscatter medium of water equivalent thickness of 2.75 g/cm^2^. Measurements can be performed with the array inserted into a dome geometry phantom named StereoPHAN (Figure 2), whose cylinder part is 15.24 cm in diameter and 20.87 cm long. The phantom can be rotated around its longitudinal axis, allowing the array orientation to be adjusted to the measurement plane chosen by the user.

To set the SRSphantom in the measurement position, it has been leveled previously using a precise digital level and has been placed in the isocenter through the treatment unit laser system. As an additional verification, CBCT images were registered with the corresponding simulation phantom CT previously acquired. The accuracy of the correspondence between both Lasers and EPID CBCT systems with the MV isocenter has been verified and adjusted by carrying out Winston‐Lutz tests by means of a ball bearing phantom placed in the accelerator radiation isocenter.

Following manufacturer recommendations,^13^ the array was initially calibrated for each treatment unit and energy in two steps. The first step includes a calibration of the relative diode response and the second is an absolute dose calibration. This calibration, which must be carried out before each measuring session, serves both to correct the absolute response of the diodes and the effect of the change in temperature.

## 3 METHOD

To perform SFRT treatment verification, a measurement method that provides adequate resolution and sensitivity must be chosen, as well as response stability under the beam configurations used to modulate the dose. Our study includes the different tests performed to determine the reliability of this equipment.

We started by verifying the general functionality of the array, checking that it complies with the characteristics described by the manufacturer in the equipment specifications. Among others, two issues should be carefully considered, first the variation in diode response with the applied dose rate and with field size, and second the variation depending on the beam incidence angle over the flat geometry of the array. Therefore, the factors applied to the measurement by the data acquisition and processing software must be verified.^14^, ^15^, ^16^, ^17^ Once these commissioning tests have been performed, a sensitivity and specificity study will be carried out to determine the array's ability to detect treatment deliverability errors. The last step in the verification will be the comparison of results for real patient dosimetries with other verification methods, such as the ionization chamber and gafchromic film.

### 3.1 Initial tests

Among general functionality tests, it has been verified that the equipment meets the manufacturer's specifications, that is, diode response homogeneity, reproducibility, repeatability, and dose linearity tests. To perform the homogeneity test, the array was placed directly on the treatment table with a 10 cm PMMA build‐up/build‐down region and irradiated with a field size beam of 10 × 10 cm^2^, larger than the sensitive area of the array, to obtain a flat enough beam. For the remaining tests, the set‐up was maintained except for the field size, which was chosen as the reference size of 5 × 5 cm^2^. To compensate for variations introduced by the linac stability, measurements were carried out with an ionization chamber located within the radiation beam, but outside the area comprising the five central diodes targeted for the tests.

#### Response correction factors

The recommendation for verification of the dose distribution applied to the rotating IMRT (VMAT) is the use of equipment whose dependence on beam incidence is negligible.^4^ In the case of a 2D array, if the beam has an oblique incidence, its path will be modified as it passes through the diode distribution and its associated electronics, notably modifying the signal collected by the detectors. Since we are going to use the equipment for verification of volumetric IMRT treatments, it is essential to verify whether the equipment is able to accurately correct orientation dependence. Variation in response due to dose rate, field size and temperature, must be compensated for by applying the corresponding correction factor. In the detector commissioning tests should include the verification of the accuracy in the application of those factors.

#### Dose per pulse (DpP) factor

The diode response depends on the pulse dose, so that as the dose rate decreases, the fraction of recombined charge carriers increases by reducing the amount of charge collected by the electrometer and therefore the diode response. This effect has been widely studied^14^ for the diodes used by Sun Nuclear in the ArcCheck, MapCheck and SRSMapCheck arrays.^14^

The correction factor applied by Sun Nuclear software compensates for the difference in response for dose rates lower than the range we used to perform the absolute dose calibration. Once the dose rate dependence has been corrected, according to the manufacturer's specifications,^13^ the variation in the response should be less than ±1.5% over the range from 100 MU/min to 2400 MU/min.

#### Beam incidence angle factor

**FIGURE 3:**
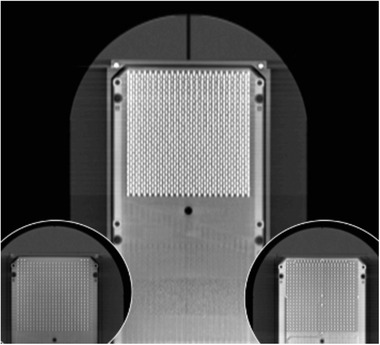
CT coronal reconstructed image that shows the diodes distribution in two PCBs. The small pictures below show the two complementary printed circuit boards (PCBs) that are overlapped and aligned in such a way that the active zone of the pn‐junction is on the same plane forming a single measurement plane

This factor has two components, the component that corrects the response variation derived from the diode geometry and the compensation from the beam attenuation crossing in its path all non‐water equivalent density components (PCB, electronics, and other diodes). Due to the way the two PCBs have been assembled, there is a difference between the individual signals collected by each PCB, which in turn varies according to the orientation of the incident beam, Figure 3. This difference in the response between PCBs is used by acquisition software to estimate the beam incidence angle and thus apply the corresponding correction factor.

The study of the beam incidence response has been carried out with different gantry angles, in such a way that the array has been oriented both parallel and perpendicular to the treatment table, to assess the response by avoiding the beam to be affected by the treatment table attenuation. To assess this effect, we have chosen the readings of the five central diodes on the longitudinal central axis of the array. Gantry value *G* = 0 deg, corresponds to normal beam incidence where no correction is applied and, *G* = 90 deg corresponds to an incidence parallel to the detector plane where several diodes and their electronic components are intersected by the beam.

#### Field size factor

Diodes present an over‐response with increasing beam field size^16^, ^17^ a behavior that is more significant for field size lower than 2 × 2 cm^2^. The SNC software allows applying a correction factor to each beamlet in the measuring movie to compensate for diode response.

The accuracy of the field size response has been analyzed, for which tests have been carried out to shape the beam in two ways, with a built‐in MLC and with a cone‐based tertiary collimation system, which has enabled us to define small field sizes accurately and reproducibly.

### 3.2 Sensitivity and specificity tests

A study has been conducted to assess the ability of the equipment to detect systematic errors that linac could introduce when delivering a treatment. For simplicity, we have separated the errors introduced into variations resulting from the deviation in the position of the leaves and derived from the accuracy of the gantry position.

Equipment sensitivity and specificity values have been estimated based on results obtained from the plans with systematic errors. Γ (2%, 2 mm) > 90% has been used as a passing threshold. As in this case, interest is focused on error detection, positive cases were those in which the dose was incorrectly delivered, whereas negative cases were those with no error in the dose administration. Sensitivity measures the fraction of incorrectly delivered plans the detector classifies as incorrect plans (positive cases). Specificity measures the fraction of calculated plans correctly delivered by the linac that are classified as correct plans (negative cases).

A total of 99 verification plans have been performed with errors induced in the size/offset of field size and angle length to reflect the detection capacity of the array. Lower and upper limits have been calculated by the Wilson^18^, ^19^ method for a 95% confidence interval, assuming normal error distribution and truncating the upper limit to a maximum value of 1.

To verify the ability of the array to detect introduced errors, the reference profile (without errors) has been compared with the profiles subject to errors acquired with the array. For simplicity, we have separated the errors introduced into variations resulting from the deviation in the position of the leaves and derived from the accuracy of the gantry position.

Leaf position errors have been generated starting from a reference prescription consisting of a narrow rectangular beam (2 × 5 cm^2^) that delivers *N* = 100 MU distributed along a CW arc of 120 deg, (gantry‐start = 300 deg; gantry‐stop = 60 deg). The position of the MLC in the reference beam *X*1 = +10 mm; *X*2 = –10 mm has been modified to introduce an offset or a deviation in field size; that is, the offset of +1 mm has been applied by modifying the position of the leaves to *X*1 = + 9 mm; *X*2 = –11 mm for an offset of –1 mm the position of the leaves changes to *X*1 = +11 mm; *X*2 = –9 mm, and so on for the rest of offsets {±2 mm, ±3 mm}. In this case, no major changes are expected in the dose delivered, but we intend to consider the ability of the equipment to detect geometric errors from the positioning of the leaves.

To increase or decrease the field size, MLC bank *X*1 position is increased/decreased {±1 mm, ±2 mm, ±3 mm} achieving the desired effect. In this respect, because it is a narrow beam, the effect on the modification of the dose delivered will be more appreciable.

The approach to errors introduced is similar to the previous case, a narrow beam of 1 × 5 cm^2^ in CW arc whose length was 60 deg (ganty‐start = 300 deg; ganty‐stop = 360 deg). For the gantry offset variation we maintained the 120 deg arc length and modified the start and end of de beam +1 deg, and therefore the gantry travel went from *G*_start _= 301 deg to *G*_stop _= 61 deg. For –1 deg offset the gantry start and end went from *G*_start _= 299 deg to *G*_stop _= 59 deg and so on for the rest of the offsets {±2 deg, ±3 deg, ±5 deg}. To increase and decrease the arc length, *G*_start_ position was increased/decreased {±1 deg, ±2 deg, ±3 deg, ±5 deg} by making again the desired effect. Table 7 provides a summary of the modified beam parameters in the specificity and sensitivity tests.

**FIGURE 4:**
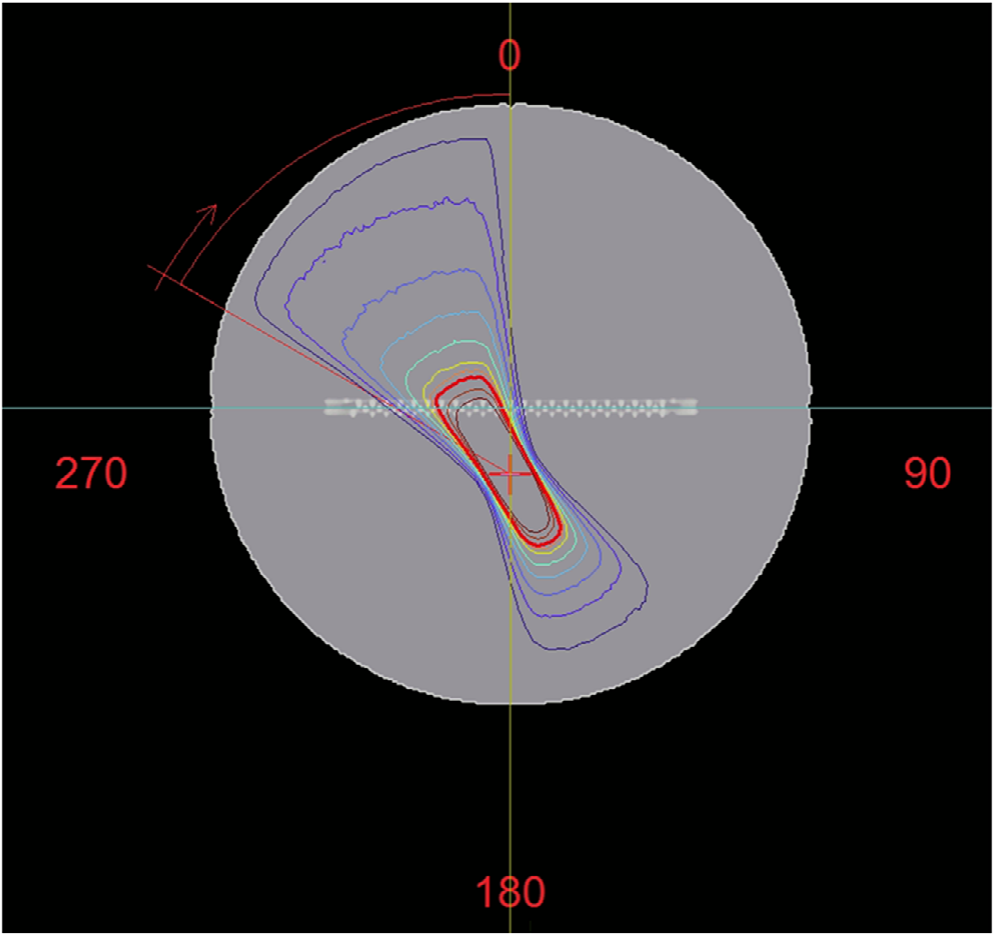
Axial CT plane of the phantom on which isodose distribution of beam proposed for the sensitivity study of variations in the arc position may be seen. It is observed how the measurement plane does not coincide with the isocenter plane and the arc has an asymmetric distribution with respect to the axis perpendicular to the detector plane

To reinforce the impact of the dose measured with the variation of the angle of the gantry, the isocenter that is, the rotation axis of the arc, has been separated from the phantom symmetry center 1.7 cm below the detector plane. The relationship between the position of the isocenter and the plane of the detector shown in an axial plane of the phantom, can be seen in Figure 4.

### 3.3 Patient specific QA for SFRT treatments

The final step of the device analysis includes the study applied to SFRT treatments for real patient cases. The patient‐specific QA protocol comprises a point measurement of absolute dose, the location of which has been placed within a low dose gradient zone, and the absolute dose distribution comparative by studying fluence maps. For each patient case, measurements have been taken on two significant planes (sagittal and coronal), trying to cover at least 3 of the multiple mini targets defined in SFRT planning.

The calculated dose matrix has been exported to the respective applications (SNC for SRSMapCheck and Omnipro for EBT3 film) for comparison with the corresponding measured matrix. The evaluation is performed qualitatively by visualizing the matching of isodose plans and quantifying by means of the function Γ (2%, 2 mm)‐threshold = 10%. Likewise, to be able to compare the three sets of values at once, (2D array, film and calculated in RTPS), ASCII files have been generated with the data needed to represent the comparative profiles using the Excel^©^ application.

## 4 RESULTS

### 4.1 Initial tests

In the homogeneity study, it has been obtained that 99.5% of detectors presented a difference in the response lower than ±0.5% and 100% of them had a difference of less than 1.0%.

In the reproducibility and repeatability tests, better results than 0.5% have been obtained. Dose linearity has been studied for a range of 5 to 600MU, the differences were lower than 0.5% for *N* > 25MU and the maximum difference was 1.7% for *N *= 5MU. Results obtained are comparable to those published by other authors for this array.^20^, ^21^

#### Response correction factors

**FIGURE 5:**
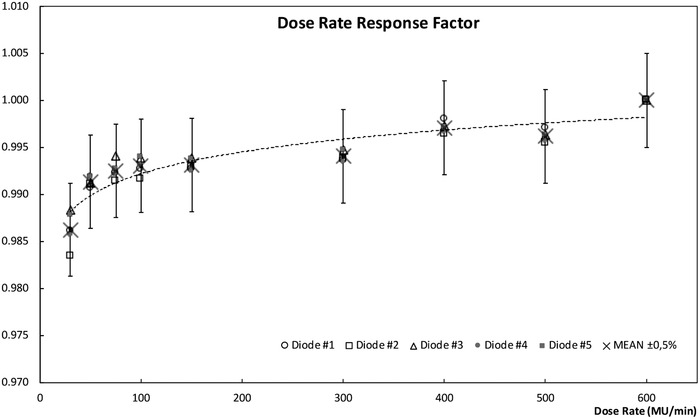
Diode response as a function of dose per pulse for a dose rate range from 30 MU/min to 600 MU/min

The results obtained for dose per pulse verification, normalized to DR_maximum _= 600 MU, agree with those published by other authors in the literature^19^, ^20^, ^21^ and they reflect a variation in diode response of up to 1.5% when the dose rate is decreased from 600 MU/min to 37.5 MU/min. In our case for DR = 30 MU/ min, the response variation obtained is better than 1.5% and for DR above 50 MU/min lower than 1%, as shown in Figure 5.

**FIGURE 6:**
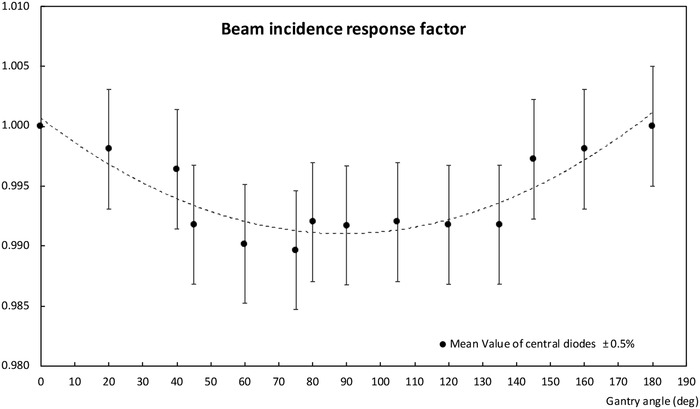
The response deviation as a function of the beam incidence shows better results than 1% for all gantry angles; where *G* = 90 deg means normal incidence at the array plane and *G* = 0–180 deg means parallel incidence

The response deviations for all beam incidences are lower than 1%, the worst results being, as expected, for normal incidence. The distribution of values according to the angle of incidence is shown in Figure 6.

**FIGURE 7:**
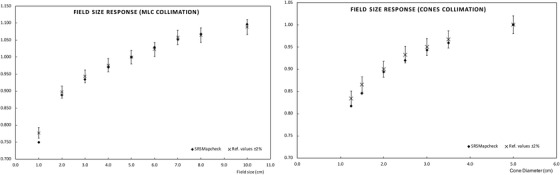
Output factors normalized to a 5 × 5 cm^2^ field size; values are compared to the linac commissioning values implemented in the RTPS. Results obtained with an MLC collimation are displayed on the left and with SRS‐cones collimation on the right

**FIGURE 8:**
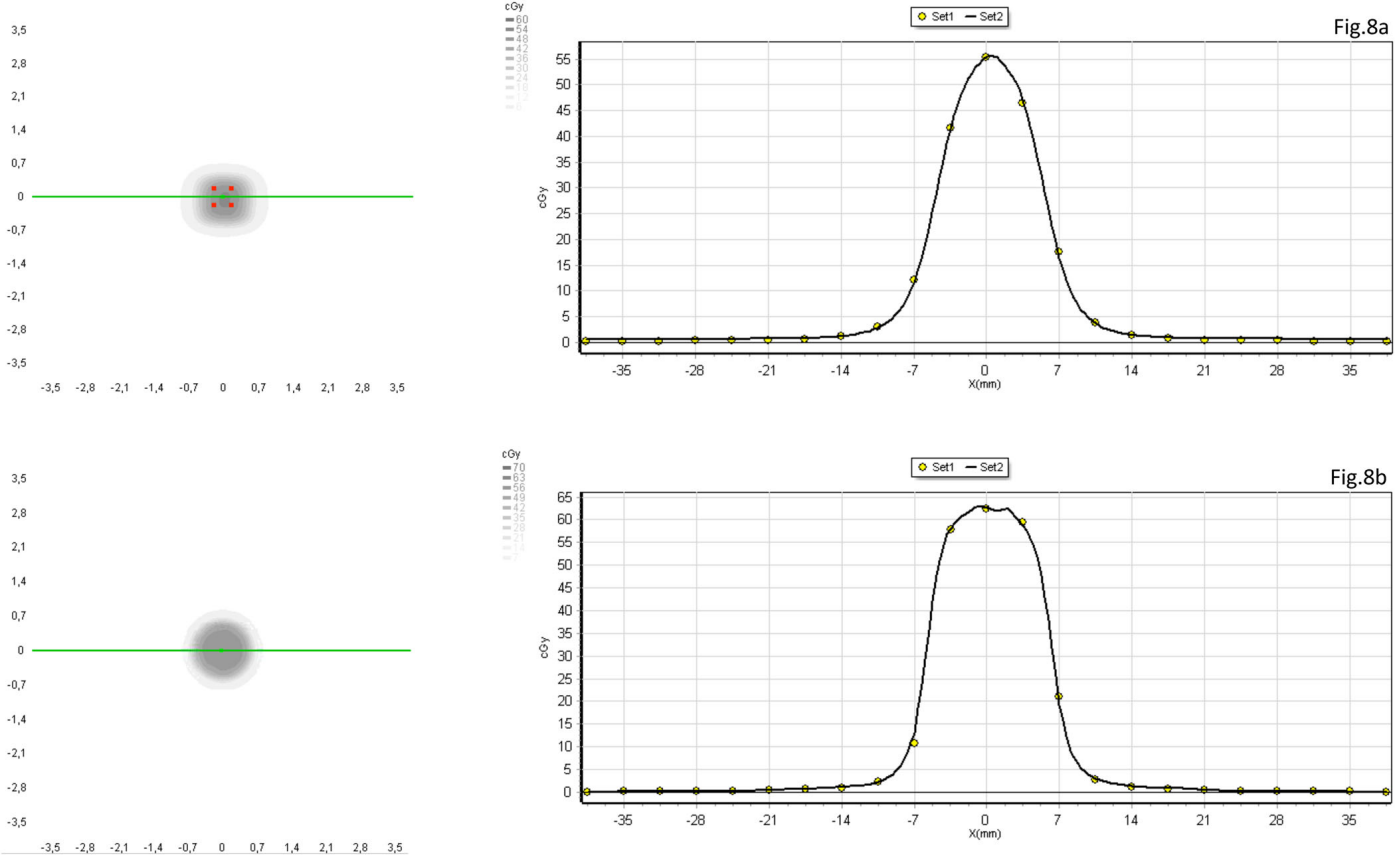
Measured profiles (dot curve) versus calculated profiles (solid line). Figure 8a shows a 1 × 1 cm2 square field size and Figure 8b cone collimation of 12.5 mm diameter

Output factor has been normalized at the reference field size, 5 × 5 cm.^2^ The values obtained from the measurements with the array have been compared to the corresponding measurements in the RTPS commissioning process, results are presented in Figure 7. Differences are within the range of ±1% for sizes from 10 × 10 cm^2^ to 2 × 2 cm^2^ for fields defined by the MLC and cones. Discrepancies start to be significant for field sizes lower than 2 × 2 cm,^2^ finding the largest discrepancy of 3.5%, for the smallest field size measured with MLC (1 × 1 cm^2^). The Gamma function, Γ (2%, 1 mm) and Γ (1%,1 mm), have been calculated to assess correspondence from the fluence planes, a threshold value of 5% has been set. The results Γ (1%,1 mm) are better than 96% for all field sizes and for both types of collimations. For Γ (2%, 1 mm) all the values are > 99.3% except for Tc 3 × 3 cm, whose value reached has been 97.1%. The results are described in more detail in Table 1. Figure 8 shows how the measured profiles faithfully reproduce those calculated, despite the limited number of detectors included in the radiation field.

**TABLE 1:** Results obtained for the gamma function calculated for different field sizes defined with MLC and cone‐based collimation

MLC Tc (cm^2^)	Γ (1%, 1 mm)	Γ (2%, 1 mm)	Cone diameter (mm)	Γ (1%, 1 mm)	Γ (2%, 1 mm)
**1 × 1**	98.2	100.0	12.5	100.0	100.0
2 × 2	100.0	100.0	15.0	100.0	100.0
3 × 3	96.5	97.1	20.0	100.0	100.0
4 × 4	99.5	100.0	25.0	100.0	100.0
5 × 5	98.6	99.9	30.0	100.0	100.0
6 × 6	96.6	99.8	35.0	96.5	99.3
7 × 7	96.0	99.7			

### 4.2 Sensitivity and specificity tests

**FIGURE 9:**
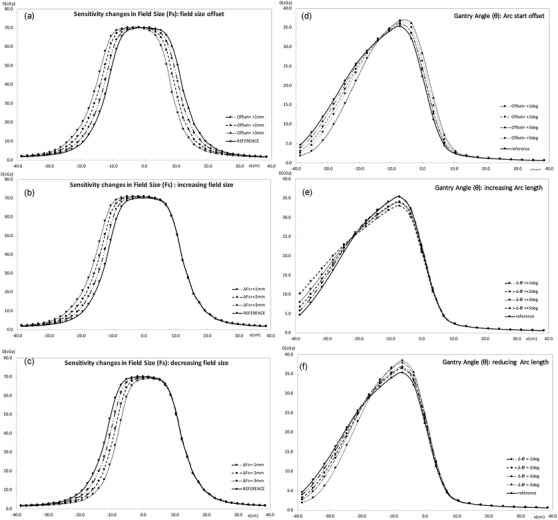
Profiles measured with the array comparing the reference beams to the beams with systematic errors introduced. (a) field size increased {0 mm, +1 mm, +2 mm, +3 mm}; (b) field size reduced {0 mm, –1 mm, –2 mm, –3 mm}; (c) offset field {0 mm, +1 mm, +2 mm, +3 mm}; (d) arc length increased {0 deg, +1 deg, +2 deg, +3 deg, +5 deg}; (e) arc length reduced {0 deg, –1 deg, –2 deg, –3 deg, –5 deg}; (f) arc start offset {0 deg, +1 deg, +2 deg, +3 deg, +5 deg}

A first approach to visualizing the array's ability to detect systematic errors has been the comparison of the measured profiles in which the resulting systematic errors have been applied with the error‐free reference profile. As it is noted in Figure 9, the profiles reproduce the expected deviations for the errors introduced.

The Gamma function Γ (2%, 1 mm) and Γ (2%, 2 mm), using threshold = 10% and global normalization, has been calculated to compare the agreement between the calculated reference beam without introducing any errors and the measured dose planes with the systematic errors. For a theoretical estimate of the expected Gamma function, the calculated reference beam has been compared with the calculated values containing the same systematic errors introduced in the measured beams.

Results, shown in Table 2, are consistent with those expected. Their values were very similar to errors derived from field size and slightly higher than the theoretical ones in the case of errors related to the gantry position. Systematic errors from variation in the position of the leaves, for a Gamma function passing threshold of 90%, present a good agreement with the expected theoretical results. However, in the case of errors in the gantry position, the measurements carried out with the array present discrepancies with theoretical results. There are values of the Gamma function obtained with the array that are above the threshold of correct treatments, while on the contrary the theoretical values would be below and would denote incorrect treatments.

**TABLE 2:** Gamma function results were obtained from the comparison of the reference field with the beams with systematic errors introduced by modifying the gantry position and field size

Field size reduced				
Γ (2%, 2 mm)			Γ (2%, 1 mm)	
Δ field size	RTPS	SRS MAPCHECK	RTPS	SRS MAPCHECK
Ref.	100.0%	100.0% (100.0–100.0)	100.0%	99.9% (99.7–100.0)
−1.0 mm	99.7%	100.0% (100.0–100.0)	66.6%	77.1% (71.1–83.5)
−2.0 mm	67.1%	71.4% (66.9–75.6)	58.0%	62.2% (60.1–62.9)
−3.0 mm	62.0%	63.2% (62.5–62.8)	54.6%	58.0% (56.1–59.8)

Field size increased				
Γ(2%, 2 mm)			Γ(2%, 1 mm)	
Δ field size	RTPS	SRS MAPCHECK	RTPS	SRS MAPCHECK
Ref.	100.0%	100.0% (100.0–100.0)	100.0%	99.8% (99.7–100.0)
+1.0 mm	99.6%	100.0% (100.0–100.0)	68.0%	78.3% (72.6–87.8)
+2.0 mm	67.6%	73.4% (68.9–77.5)	58.8%	64.6% (63.5–65.7)
+3.0 mm	61.4%	64.8% (64.8–64.8)	54.4%	60.5% (60.0–61.0)

Field size Offset				
Γ(2%, 2 mm)			Γ(2%, 1 mm)	
Offset	RTPS	SRS MAPCHECK	RTPS	SRS MAPCHECK
Ref.	100.0%	100.0% (100.0–100.0)	100.0%	99.9% (99.7–100.0)
+1.0 mm	99.6%	100.0% (100.0–100.0)	38.8%	45.5% (44.6–46.6)
+2.0 mm	40.0%	41.5% (41.6–41.4)	29.6%	32.2% (31.5–33.2)
+3.0 mm	30.6%	32.9% (32.9–32.8)	24.1%	26.6% (25.8–27.3)

Arc reduced				
Γ(2%,2 mm)			Γ(2%,1 mm)	
Δ arc	RTPS	SRS MAPCHECK	RTPS	SRS MAPCHECK
Ref.	100.0%	99.9% (99.7–100.0)	100.0%	98.5% (97.0–99.5)
–1.0 deg	99.8%	98.9% (98.2–99.5)	78.2%	92.6% (90.5–94.6)
–2.0 deg	82.7%	92.3% (91.1–94.3)	57.9%	68.1% (65.2–72.1)
–3.0 deg	67.8%	73.8% (72.3–75.3)	45.7%	48.5% (48.5–48.5)
–5.0 deg	55.8%	53.7% (53.7–53.7)	23.4%	32.0% (32.0–32.0)

Arc increased				
Γ(2%,2 mm)			Γ(2%,1 mm)	
Δ arc	RTPS	SRS MAPCHECK	RTPS	SRS MAPCHECK
Ref.	100.0%	100.0% (99.8–100.0)	100.0%	98.6% (97.8–99.5)
+1.0 deg	99.6%	99.9% (99.8–100.0)	81.6%	85.9% (84.0–87.5)
+2.0 deg	84.8%	90.0% (88.6–91.9)	61.5%	66.6% (64.3–68.9)
+3.0 deg	70.6%	77.2% (75.9–78.4)	50.9%	61.0% (59.8–62.1)
+5.0 deg	54.0%	62.8% (62.8–62.8)	38.9%	50.4 %(50.4–50.4)

Offset Arc				
Γ(2%,2 mm)			Γ(2%,1 mm)	
Offset	RTPS	SRS MAPCHECK	RTPS	SRS MAPCHECK
Ref.	100.0%	99.9% (99.5–100.0)	100.0%	98.4% (96.2–99.5)
1.0 deg	99.8%	99.7% (99.0–100.0)	78.2%	86.1% (80.3–91.9)
2.0 deg	82.7%	90.3% (86.6–98.7)	57.9%	50.7% (45.7–54.9)
3.0 deg	67.8%	70.3% (68.1–72.8)	45.7%	36.2% (35.4–36.9)
5.0 deg	55.8%	53.7% (42.9–59.5)	23.4%	29.5% (27.6–31.4)

*Note*: The expected theoretical results (RTPS) are shown with the SRSMapCheck measured results.

It is noted that treatments with systematic errors of 2 deg, which in theory should be marked as incorrect, pass the filter of Γ (2%, 2mm) > 90%. Similarly, for Γ (2%,1mm), where values with errors of Δ*θ* = 1 deg, which theoretically present values of less than 90%, produced a favorable result of the Gamma function, with values of Γ (2%, 1 mm) > 90%.

Table 3 shows how the fraction of the correctly classified plans increases by changing the metric of the gamma function. Sensitivity, that is the ability to detect the plans incorrectly imparted reliably (lower limit) increased from 0.65 to 0.86. On the other hand, specificity (lower limit), this is the correct plans properly classified, decreased from 0.92 to 0.85 when more strict criteria were applied. The results of the previous tests have been broken down according to the type of error induced. Statistical results for a 95% confidence level are shown in Tables 4 and 5 for errors derived from the position of the MLC and gantry, respectively.

**TABLE 3:** Summary of sensitivity and specificity obtained from test treatment plans with systematic errors derived from field size and gantry position

*N* = 99	Fraction of correctly classified plans			Sensitivity			Specificity		
(IC = 95%)	Value	Lower limit	Upper limit	Value	Lower limit	Upper limit	Value	Lower limit	Upper limit
Γ (2%, 2 mm)	0.92	0.84	0.96	0.81	0.65	0.91	1.00	0.92	1.00
Γ (2%, 1 mm)	0.96	0.89	0.99	0.94	0.86	0.98	1.00	0.85	1.00

**TABLE 4:** Sensitivity, specificity and F1 score results for systematic error tests of the MLC position

MLC position errors	Sensitivity			Specificity			F1‐Score
(*N* = 48, IC = 95%)	Value	Lower limit	Upper limit	Value	Lower limit	Upper limit	Value
Γ (2%, 2 mm)	1.00	0.78	1.00	1.00	0.86	1.00	1.00
Γ (2%, 1 mm)	1.00	0.70	1.00	1.00	0.87	1.00	1.00

**TABLE 5:** Sensitivity, specificity and F1‐score results for systematic error tests of the Gantry position

Gantry position errors	Sensitivity			Specificity			F1 score
(*N* = 51, IC = 95%)	Value	Lower limit	Upper limit	Value	Lower limit	Upper limit	Value
Γ (2%, 2 mm)	0.67	0.47	0.84	1.00	0.85	1.00	0.80
Γ (2%, 1 mm)	0.89	0.73	0.96	1.00	0.75	1.00	0.94

The change of metric in the gamma function to evaluate the plans from 2 to 1 mm does not seem to induce improvements in the identification of correct or wrong plans when the error comes from the positioning of the leaves (e.g., SFRT with IMRT). When errors are introduced into the gantry angles, the change in the metric of the GI to more restrictive parameters, achieves an improvement in detectability.

Using the Gamma function metric Γ (2%, 2 mm) and an inferior limit value of 90%, the detector has a high specificity, while its sensitivity has a moderate mean value of 81%. Sensitivity values increase significantly, above 86%, when applying a Gamma function Γ (2%, 1 mm). In a few words, considering the fraction of correctly classified plans, using the most restrictive metric (at the expense of losing specificity) more than 95% of verified plans were successful. These values confirm the ability of this array to detect misalignments and dose errors in radiotherapy treatment plans. So, to improve the sensitivity of the array in the case of SFRT treatments, by means of VMAT treatment techniques, the use of Γ (2%, 1 mm) is recommended instead of Γ (2%, 2 mm).^4^, ^7^

### 4.3 Patient specific QA for SFRT treatments

**FIGURE 10:**
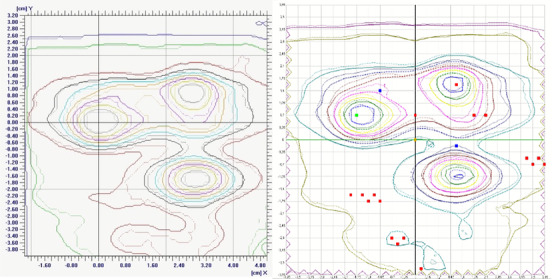
Fluence map comparison of a coronal plane. Isodose agreement for EBT3 measured (solid line) versus RTPS (dot line) show on the left side and SRSMapCheck measured (solid line) versus RTPS (dot line) on the right side

To better illustrate the process followed, we will begin by analyzing an example of the results obtained in one patient. The agreement of absolute dose distribution was carried out by merging the measured and calculated fluence maps of a representative coronal plane, this is shown in Figure 10.

**FIGURE 11:**
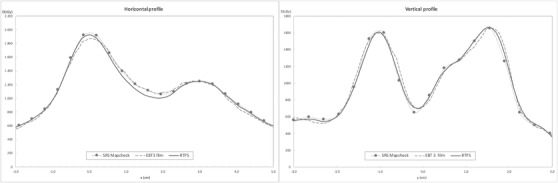
Horizontal (left) and vertical (right) profiles comparing the results of measures with gafchromic film, SRSMapCheck, and Monaco RTPS. Profiles are taken over the dose high dose gradient regions that conform to the dose vertices in SFRT treatments

Horizontal and vertical profiles have been compared to further analyze where differences appear with respect to the dose calculated values, and between both measurement methods. Two planes have been chosen, one horizontal (Figure 11, left) and the other vertical (Figure 11, right) taken over the largest gradient areas of the coronal plane represented above.

When analyzing in more detail the results obtained with both methods, especially in overlapping dose profiles, we can observe slight discrepancies in both maximum peak and abrupt minimum areas. In such a way that there is a better match between the measurement with the array and the calculation of the maximums, despite the lower resolution of the array, to that obtained with the film. It is therefore observed that in the maximum dose area, the value obtained with the film is lower than that calculated or obtained with the array. This fact is because the dose levels delivered are within the dynamic range, but outside the optimal response range of this type of gafchromic film. Instead, in the valley area the dose collected by both measuring systems and therefore the one we can consider as the delivered dose by the linac, is slightly higher than the calculated, a discrepancy that we can attribute to the high degree of modulation and complexity required by the SFRT technique.

The global study of actual cases comprised 17 patients, distributed into 11 lung tumors, 3 gynecological and 3 soft tissue tumors, (one inside of the oral cavity).

As shown in Table 6, all point measurements performed with IC showed difference values better than 3.5%. compared to the calculated ones. Γ (2%, 2 mm) obtained for the measurements carried out with the gafchromic film showed a lower pass rate with an average value of 94.1% compared to the 98.6% reached by the measurements with the array. The pass rate obtained with the SRSMapCheck Array for all patients has been better than 95.9%, while with the gafchromic film the minimum value achieved was 87.2%.

**TABLE 6:** Results of the specific patient QA performed at 17 SFRT treatments

	Γ (2%, 2 mm)		%Diff.
	SRSMapCheck	EBT3 film	Pinpoint I.C.
Mean	98.8%	93.8%	0.6%
Max	99.9%	98.6%	2.2%
Min	95.9%	87.2%	–1.7%
%σ	1.0%	3.4%	1.3%

The gafchromic film processing has associated greater uncertainty due to both the calibration process and the manual registration of isodose planes. In particular, this last step of manual registration of the scanned image, based on fiducial points, is less accurate and cannot compete in accuracy and reproducibility with the measuring process followed by the array and the phantom, a fact that can be clearly noticed when comparing the overlapping of the isodose planes made with both measurement methods.

## 5 DISCUSSION

To modulate the dose and generate multiple mini‐targets that conform to the vertices of SFRT treatment, a high number of MU, several small‐sized and complex conforming beamlets are applied, all these elements are associated with a high Modulation Complexity Score (MCS).^22^, ^23^ Therefore, the spatial resolution of the array plays a key role when choosing the appropriate detector to carry out this kind of verification. In this regard, studies by several authors have been published,^24^, ^25^ analyzing the maximum distance between detectors allowable to measure and detect deviations in high‐gradient IMRT dose distributions. These works, based on the application of the Nyquist Theorem, demonstrate that the use of a spatial sampling frequency of 0.4 mm^−1^, corresponding to a spacing between detectors of 2.5 mm, is appropriate to detect errors in positioning of 1 mm or more. The small size of detectors that form the array does not show a convolution‐type influence, allowing them to reproduce profiles with high gradient levels in a reliable way. Our study shows examples, reflected in Figures 8 and 9, where dose profiles for small field sizes with systematic errors are shown, and Figure 11, in which profiles taken over the largest gradient areas are compared. Otherwise, it should not be forgotten that the aim of the equipment is not the descriptive acquisition of high‐resolution dose profiles, but rather the detection of errors when comparing those profiles with the planned dose distribution.

We have analyzed the sensitivity to detect errors introduced by using narrow beams of 1 cm in width, whose measurement has been compared to the values obtained either theoretically by dosimetries calculated with RTPS, or to other high‐resolution measurement system, gafchromic film, whose response has been widely contrasted. It should be noted that our systematic error detection study was conducted in simple dose distributions, so that the results obtained for the agreement index, such as Gamma function or sensitivity and specificity, do not reflect the complexity of actual clinical treatments.

## 6 CONCLUSIONS

In this paper we have described the tests to verify a high spatial resolution 2D array, model SRSMapCheck (Sun Nuclear Corp., Melbourne, FL), reliability, specificity and sensitivity to detect misalignments and other dosimetric errors.

Comparisons with gafchromic measurements and with the calculated theoretical value have shown that the agreement index, Gamma function, obtained with SRSMapCheck array always presented a higher value, resulting in a greater number of plans considered correct. Generally, the specificity of the detector was good, and therefore it properly identified the correct plans (using Γ (2%, 2 mm) as the evaluation metric), regardless of the source of these errors (MLC or gantry position). However, to achieve good sensitivity and ensure that incorrect plans are not classified as correct, it required using a more restrictive metric for VMAT type treatments where the errors may come from a combination of leaf positions and gantry positions. Sensitivity can thus be improved either by increasing the acceptance threshold of the Gamma function (from 90% to 95%) or using a metric of Γ (2%,1 mm). These results are in line with the proposals published in papers by other authors.^4^, ^7^

Consequently, we tested the SRSMapCheck array response under different irradiation conditions, after having characterized the device's sensitivity and specificity. We found that the characteristics of the equipment together with the correction factors applied, let us to reliably perform patient specific QA for a wide range of complex treatments, not only SRS treatments but also focused on treatments that include multiple targets treated at once and high gradient dose plans such as those achieved in SFRT.

The methodology and results presented above, although exemplified in the specific case of the SRSMapCheck, will be useful in advancing the standardization of QA protocols necessary to establish the reliability limits of 2D arrays in SFRT and other spatially complex treatments, both related to the array resolution and to the variation of the response of each individual diode to steep gradient dose field.

## CONFLICT OF INTEREST STATEMENT

The authors have no conflicts of interest to disclose.

## AUTHOR CONTRIBUTION STATEMENT

Luis M. Larrea was the physician in charge of SFRT patient treatments. Patricia Gil and Jose D. Lago‐Martin participated in performing and collecting the data necessary for the research; Jose D. Lago‐Martin performed the statistical data analysis and Maria C. Banos‐Capilla designed the tests performed, analyzed the data and wrote the paper; and all authors checked the final version of the manuscript.

## Data Availability

The data that support the findings of this study are available from the corresponding author upon reasonable request.

## 1

Summary of modified beam parameters in specificity and sensitivity tests for treatment plans generated with systematic errors introduced in field size and gantry position

**Table:**

	*Y*2 (mm)	*Y*1 (mm)	Field width (mm)
*Reference*	*10*	*10*	*20*
+1 mm offset	11	9	20
+2 mm offset	12	8	20
+3 mm offset	13	7	20
+1 mm	11	10	21
+2 mm	12	10	22
+3 mm	13	10	23
–1 mm	9	10	19
–2 mm	8	10	18
–3 mm	7	10	17

	Gantry start (deg)	Gantry end (deg)	Arc length (deg)
*Reference*	*300*	*360*	*60*
1 deg offset	301	359	60
2 deg offset	302	358	60
3 deg offset	303	357	60
5 deg offset	305	355	60
+1 deg arc	299	360	61
+2 deg arc	298	360	62
+3 deg arc	297	360	63
+5 deg arc	295	360	65
–1 deg arc	301	360	59
–2 deg arc	302	360	58
–3 deg arc	303	360	57
–5 deg arc	305	360	55
